# Allometric analysis of a morphological anti-predator trait in geographic populations of Japanese crucian carp

**DOI:** 10.1038/srep41943

**Published:** 2017-02-02

**Authors:** Sakie Kodama, Hiroka Fujimori, Hiroshi Hakoyama

**Affiliations:** 1 Department of Nature and Environment, the Open University of Japan, Chiba, 261–8586, Japan; 2 National Research Institute of Fisheries Science, 1088 Komaki, Ueda, Nagano, 386–0031, Japan

## Abstract

Costly anti-predator traits tend to be expressed only in high-predation conditions. For the cyprinid fish genus *Carassius*, deeper body depth is more adaptive to avoid predation by gape-limited piscivorous fish, but it raises swimming costs. It is therefore predicted that the relative body depth will decrease when the prey fish has reached a size larger than the predator gape-size. This prediction was tested by allometric analysis of the relation between body depth and standard length of triploid asexual females of the Japanese crucian carp (*Carassius auratus* sspp.) sampled from 13 geographic populations. The overall allometric relation was not significantly different from isometry. The estimate of the common major-axis slope was close to 1 (near-isometry). The mean relative body depth differed significantly among populations. A significant positive correlation was found with the mean annual air temperature. The geographic variation suggests that local selection pressures vary. In conclusion, the hypothesis that larger fish will have lower body depth was not supported, perhaps indicating that deep body depth in large fish is adaptive for some reason other than defense against piscivorous fish.

Defense traits against predation often entail costs or tradeoffs^1^,^2^,^3^. Therefore, natural selection favors efficient expression of defense traits depending on the level of predation risk that might vary in both unpredictable and predictable ways^2^. Predator-inducible defenses that can be induced by cues from predators cope with the unpredictable variation of predation risk^2^,^3^,^4^. For the crucian carp (*Carassius carassius*), a well-studied freshwater fish for testing the theory of predator-inducible defense, the presence of chemical cues from piscivorous fish (northern pike, *Esox lucius*) has been shown to induce a deeper body depth^5^,^6^. The predator-induced morphology of crucian carp can be regarded as a beneficial defensive trait against gape-limited piscivorous fish. Experimental reports have described that the body depth of crucian carp relative to the mouth size of pike limits its maximum prey size^5^,^7^. Actually, pike prefer to feed on shallow-bodied crucian carp^8^. Deep-bodied crucian carp possess enhanced escape performance^9^. Moreover, the induced deeper-bodied morphology has been shown to be correlated with lower activity, which can be regarded as an anti-predator trait of prey species^10^,^11^. However, the deeper body morphology of crucian carp imposes costs in terms of greater hydrodynamic drag^12^ and lower ability of resource competition (a lower growth rate in enclosures with high-density zooplankton^13^, and lower foraging efficiency on zooplankton^14^). Therefore, the predator-inducible defense of crucian carp is explainable as a cost-saving strategy in unpredictable environments^2^,^5^.

Size-limited predation can also cause changes in the extent of constitutive or induced defense during the ontogenetic growth of an organism (“allometry of defense”)^15^. The allometry of defense deals with the predictable change of predation risk during the animal’s life span. For crucian carp, when an individual has grown to the absolute size of refuge from predation, the expression of the costly deeper-bodied morphology is expected to be reduced^5^. The prediction that the body depth of large crucian carp has a lower growth rate than the body length (defense-allometry hypothesis; negative allometry^16^,^17^ in body depth) was tested using linear regression analysis in five ponds holding piscivorous fish: three Finnish ponds of Patsonlampi pond *n* = 16, Kivilampi pond *n* = 24, Marjalanlampi pond *n* = 13^18^ two Swedish ponds of Severln’s pond and Mats’ pond^5^. The results were positive for the three Finnish ponds, but were not positive for the two Swedish ponds^5^. Heterogeneity of results might be explained by unknown environmental differences among ponds (e.g., food availability, temperature) or by the small sample size. In a meta-analysis context, studies with a small sample size are known to yield differing results sometimes, even when the studies share a common effect size. The defense-allometry hypothesis for the body-depth morphology of crucian carp is therefore not fully understood yet.

For this study, we test the defense-allometry hypothesis for body-depth morphology using geographic populations of Japanese crucian carp (*Carassius auratus* sspp.). Japanese crucian carp, which are closely related to crucian carp, are distributed widely throughout freshwater areas in Japan^19^ with a sympatric piscivore, the Japanese common catfish (*Silurus asotus*)^20^. Results of an experimental study demonstrated that the presence of alarm cues from injured conspecifics and chemical cues from pike increase the body depth and body weight of domesticated goldfish (*Carassius auratus auratus*). The enlarged morphology reduces predation risk from a piscivorous fish (yellow perch *Perca flavescens*)^21^. The presence of Japanese common catfish also induced deeper-bodied morphology of Japanese crucian carp in an experimental study (Kodama & Hakoyama, manuscript in preparation). Therefore, the Japanese crucian carp is a species that would also be appropriate for testing of the hypothesis of the allometry of defense. We study natural triploid asexual females^19^,^22^,^23^,^24^ to exclude potential effects of sex and ploidy level on the allometry of defense.

For the analysis conducted using the allometric equation *Y* = *αX*^*β*^ (*X* = size, standard body length; *Y* = shape, body depth)^16^,^17^, ordinary linear regression was found to be unsuitable to estimate the logarithmic linear functional relation between size and shape log*Y* = log *α* + *β* log *X* or to test for a non-one allometric slope *β* because both variables are expected to be subject to natural variation and measurement error^25^. Therefore, if we apply linear regression analysis, then the slope estimate is biased downward^25^. When both variables are measured in the same units, as is true in the morphometry, the major-axis regression is a recommended method to estimate and to test the logarithmic linear functional relation in analyses of allometry^25^,^26^. The major-axis regression depends on an assumption that the amount of the residual variance of two variables is equal^27^. Instrumental-variable regression (also known as “the covariance ratio method”^27^) is another recommended method that does not assume equal residuals, but which requires a third measurement of the “instrumental” variable (another morphological trait)^27^,^28^. We use both major-axis and instrumental-variable regressions for logarithmic line-fitting in allometry analyses of the body depth of Japanese crucian carp.

## Results

### Allometric relations between body depth and standard length

**Figure 1:**
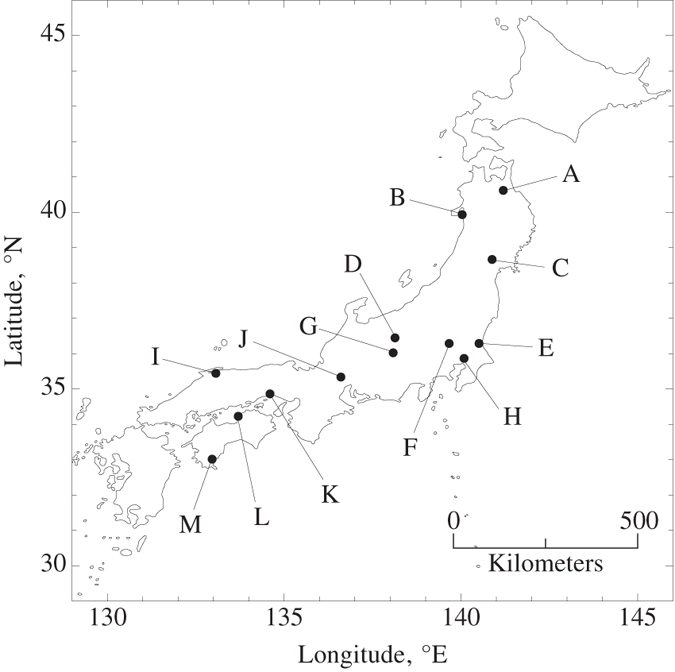
Map of Japan showing geographical locations of the studied populations of Japanese crucian carp. The piscivorous Japanese common catfish co-occurs in the same habitats. Geographic information for the populations is presented in Table 1. The map was constructed using Mathematica ver. 9.0 (Wolfram Research, Inc., URL http://www.wolfram.com/mathematica/).

**Figure 2:**
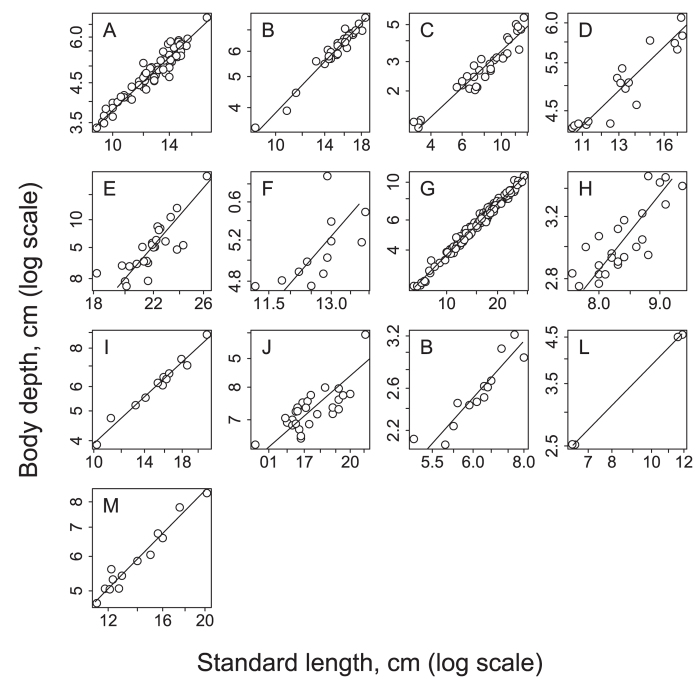
Allometric relations between standard length and body depth of the triploid asexual Japanese crucian carp from 13 populations. Population labels follow Fig. 1. Each point is an individual fish. Solid lines are major axis regression lines of log body depth on log standard length for the populations.

A double logarithmic plot of body depth on standard length of the triploid asexual Japanese crucian carp from 13 populations (Fig. 1, Table 1) shows a linear relation (Fig. 2). The strengths of the linear relation (*R*^2^) were generally high but were variable among the populations (major axis regressions, *R*^2^ = 0.402 to 0.999; Table 2). The difference in allometric slope among populations was not significant (*H*_0_: slopes of the major axis regressions are equal: likelihood ratio statistic 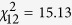, *P* = 0.23; Table 2). The overall allometric relation was not significantly different from isometry (*H*_0_: The common major-axis slope is 1: likelihood ratio statistic 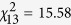, *P* = 0.27). The common major-axis slope was estimated as 1.004 (95% CI, 0.989–1.026), indicating a nearly isometric growth pattern (i.e., *Y* = *a*_*i*_*X* + [residuals term] for the *i*-th population, where *X* = standard length, *Y* = body depth, and *a*_*i*_ = constants). This isometric property enables us to use the relative body depth (body depth – length ratio, *Y/X*) in the following analysis of the pattern of the trait variation among populations because the ratio is independent of body size in cases of isometry^25^,^29^. Both the major axis and instrumental variable methods yield similar estimates and 95% confidence intervals of the allometric slope for each population (Table 2).

### Between-population and within-population variation of relative body depth

**Figure 3:**
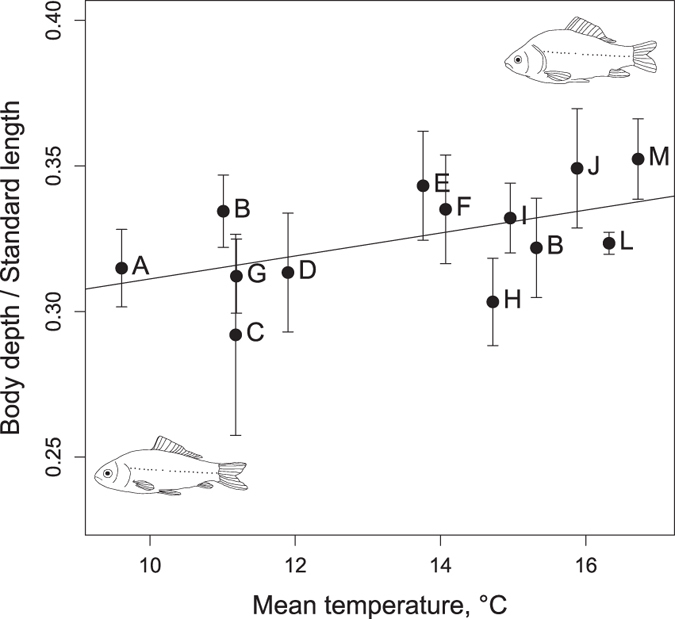
Relative body depth (body depth/standard length) of the triploid asexual Japanese crucian carp as a function of the 30-year average of mean annual air temperature. Each symbol and bar denotes the mean trait value and SD per population. Population labels follow Fig. 1. The solid line is a regression line from the linear mixed effects model (*y* = 0.004*x* + 0.27, see the text for the model). An average fish from the lowest mean trait population (C) and highest mean trait population (M) are presented to show the magnitude of trait variation.

The mean relative body depth of the triploid asexual Japanese crucian carp differed significantly among 13 populations (Fig. 3, Welch’s one-way ANOVA weighted for unequal variances, *F*_12,67.08_ = 26048, *P* < 2.2 × 10^−16^). The total variation in the relative body depth was 45% explained by the between-population variation and 55% by within-population variation (Kulinskaya and Staudte’s weighted coefficient of determination, *ρ*^2^ = 0.45). Within-population variation in the relative body depth was significantly different among populations (Fig. 3, Levene’s test of homogeneity of variances: *F*_12,420_ = 12.48, *P* < 2.2 × 10).

### Climate and relative body depth

The relative body depth of the triploid asexual Japanese crucian carp increased significantly with the 30-year average of mean annual air temperature (Fig. 3, linear mixed-effects model with a heteroscedastic random effect for each population, *t* = 2.22, *P* = 0.0485). The mean air temperature explains 15% of the total variation in the relative body depth (Nakagawa and Schielzeth’s 
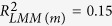
, which can be interpreted as a medium effect size^30^).

## Discussion

The absolute size refuge from catfish predation (ca. 7 cm body depth estimated in Methods: pilot study 2) was included in the range of body depths of the studied carp (1.2–11.8 cm; Fig. 2). Therefore, the defense-allometry hypothesis predicts that the body depth of the large carp (>7 cm in standard depth) has a lower growth rate than the body length to reduce the expression of the costly anti-predator morphology. However, contrary to that prediction, results show that the allometric relation estimated by the major-axis and instrumental-variable regression was almost isometric for triploid Japanese crucian carp in in 13 predator-sympatric populations. Some costs of negative relative growth might outweigh its benefits and/or some constraints might limit the optimal growth^31^. One predation-based explanation for this finding is that the high body depth of large carp is still an adaptive anti-predator trait to avoid predation from large piscivorous birds. Especially, a diving, gape-limited^32^ piscivorous bird, the great cormorant *Phalacrocorax carbo*, which is commonly distributed throughout Japan^33^, is able to feed on large fish (greater than 50 cm standard length) of numerous species^34^ including Japanese crucian carp^35^. The ontogenetic growth of Japanese crucian carp can therefore cause a shift in predation pressure from piscivorous fish to piscivorous birds. If so, large Japanese crucian carp that reached the size refuge from catfish predation would still express the costly deep body depth to resist predation from gape-limited piscivorous birds. An alternative explanation is that the deep body shape of Japanese crucian carp might be adaptive, irrespective of body size, in a foraging context. In structurally complex habitats requiring greatest control of maneuver, a deep laterally flattened body is maneuverable and adaptive to consume non-evasive prey^36^. For example, deep-bodied fish living in littoral zones have been shown to have higher foraging efficiency on benthic prey than shallow-bodied fish living in open water. Moreover, shallow-bodied fish are superior to deep-bodied fish in foraging on plankton^37^,^38^. Indeed, Japanese crucian carp live in vegetated lakes, ponds, and slow-moving rivers, searching and feeding on zooplankton, aquatic insects, snails, and plant debris as a feeding generalist (see some earlier reports describing food habits^39^,^40^). A field report has described that zooplankton are preferred only by the smallest size class of crucian carp (<3 cm TL). Benthic prey represent the major part of the diet for other large size classes^41^. The deep body shape of Japanese crucian carp might therefore be maneuverable and beneficial for foraging on benthic prey in the complex-structured aquatic vegetation. This explanation is apparently not inconsistent with a result presented in the Introduction that crucian carp with deeper body depth have lower ability of food competition in homogeneous enclosures with zooplankton^13^.

Our results also show that a significant difference exists in relative body depth among the populations of triploid Japanese crucian carp. A geographic cline exists for the relative body depth along a temperature gradient. For the warmer climate, the body depth tended to be deeper, and the effect size was medium. Some morphological adaptation to the local environments is expected to occur along a geographic gradient. The geographic cline in relative body depth might be explained by a geographic gradient of predation pressure, as suggested for morphological variation in stickleback populations^42^. Predation pressure of piscivorous fishes can decrease along with decreasing temperature^43^. Indeed, the activity (foraging activity) and its duration of Japanese common catfish have been shown to decrease at lower water temperatures of 10–25 °C^44^. The abundance of great cormorants has been shown to be low in northern habitats (including populations A, B, and C in Fig. 1), but high in other regions, especially in the Kanto (including populations E, F, and H) and Kinki regions (including population J)^45^. Another possible explanation for the body-depth cline is that food availability increases along with increasing temperature. In addition, higher resource availability (prey density and composition) causes the deeper body depth of Japanese crucian carp. The effect of food level on body depth is known for crucian carp and goldfish: fishes with high food levels have deeper body depth than those with a low food level^5^,^21^. Diet-induced morphological expression has been found for crucian carp (deep body depth with benthic prey and low body depth with planktonic prey)^14^.

In conclusion, results show nearly isometric growth in the body depth of Japanese crucian carp. Intraspecific variation is evident in the relative body depth of Japanese crucian carp under natural conditions, as are between-population and within-population variation, irrespective of whether it is constitutive or induced. The geographic variation suggests historical isolation and morphological adaptation to local environments, particularly adaptation to environmental factors related to temperature variation along a geographic gradient.

## Methods

### Ethics statement

All fieldwork was performed in accordance with local ethical regulations and agreements. All animal procedures followed strict guidelines set forward by the Japan Fisheries Research and Education Agency (FRA) and were approved by the FRA.

### Fish collections

Fish samples were collected from 13 geographic populations in Japan (Fig. 1; Table 1) during December 2009–June 2012 by collaborators with trap nets, gill nets, and cast nets. Either living or frozen samples were dispatched to the laboratory for analysis. Frozen samples were stored at −30 °C before analysis. Japanese common catfish co-occur in the same habitats.

### DNA ploidy measurements

The populations of Japanese crucian carp include sexual and asexual fish, which are distinguishable by ploidy or cell size^19^,^22^,^23^,^24^. Triploid carp are fundamentally all female; they reproduce gynogenetically^22^,^23^,^24^. To simplify the analysis and to exclude potential effects of DNA ploidy level and sex on body depth, we studied triploid asexual individuals. The ploidy level of carp was determined using flow cytometry (see Method S1 for details).

### Morphometrical measurements

The standard length (tip of snout to end of hypural plate), body depth (maximum depth of body at front of the dorsal fin origin), and the caudal peduncle depth (least depth of the caudal peduncle) of the samples of carp were measured to the nearest millimeter. The caudal peduncle depth was measured as an instrumental variable for the instrumental-variable regression^27^. Before morphometrical measurements, living fish were anesthetized with 0.2 ml/L 2-phenoxyethanol. Then frozen fish were thawed at room temperature. Sex of mature living fish was discerned by gentle pressure on the abdomen: males excrete sperm; females excrete eggs^23^. Sex of the frozen samples of mature fish was discriminated by observing their gonads. Sex of some immature fish could not be determined reliably. We confirmed that most of the triploid samples were female (98% of 378 individuals) as reported from earlier studies^22^,^23^,^24^. Therefore, we used both the triploid females and minor unsexed triploid individuals for analyses.

### Temperature of geographic regions

Temperature data for each region (population) were obtained to assess the relation between temperature and body depth of carp. Mean annual air temperature of geographical regions listed in Table 1 for 1984–2013 was obtained from a climate database of Japan (Japan Meteorological Agency). Temperature data were obtained from the nearest meteorological station to each region (A, Towada; B, Oga; C, Kashima-dai; D, Ueda; E, Mito; F, Oyama; G, Suwa; H, Abiko; I, Matsue; J, Ōgaki; K, Himeji; L, Tadotsu; M, Suzaki). We found additional published information related to some environments (see Table S1), but these were not used because there were too many missing data. No quantitative data related to predation pressure and food abundance are available. Only air temperature data were available for analysis.

### Statistical analysis

The allometric equation^16^,^17^ *Y* = *αX*^*β*^ and the logarithmic linear form log *Y* = log *α *+ *β* log *X* were used as a model to study the functional relation between body depth *Y* and standard length *X* of carp. *β* is the allometric coefficient (slope) that expresses the relative growth rate. Relative growth of the body depth might be positive (*β* > 1), negative (*β* > 1), or isometric (*β* = 1). Because both body depth and standard length are expected to be subject to errors and because both variables are measured in the same units (cm), major-axis regression was used to estimate the allometric equation^25^,^26^,^27^. The R package smart^46^ was used for analysis of populations of carp to test the hypotheses: the common major-axis slope is 1; slopes of the major axis regressions are equal. The package was also used to estimate the common allometric slope of the major-axis regression^26^ (see Supplementary Code S1). Instrumental-variable regression was also performed to estimate the allometric slope of each population^27^,^28^. The caudal peduncle depth was used as the instrumental variable. The anderson.rubin.ci function in the R package ivpack^47^ was used to estimate the allometric slope and the 95% confidence intervals of instrumental-variable regression (see Supplementary Code S2). Welch’s one-way ANOVA weighted for unequal variances^48^ was applied to compare the relative body depth of populations using the oneway.test function in R. Kulinskaya and Staudte’s weighted coefficient of determination^49^ was used to estimate the effect size of Welch’s one-way ANOVA. Levene’s test was used to test the homogeneity of the variances of relative body depth among populations using the Levene Test function in the R package car^50^. Linear regression analysis with heteroscedastic errors (a linear mixed-effects model with a heteroscedastic random effect for each population)^51^ was performed to test the relation between air temperature and relative body depth using the lme function in the R package nlme^52^. The heteroscedastic regression model was better than the corresponding homoscedastic model by likelihood ratio test (shown only in Supplementary Code S3). Nakagawa and Schielzeth’s 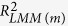
53 was used to estimate the effect size of the linear mixed-effects model using the r.squaredGLMM function in the R package MuMIn^54^. A histogram of the relative body depth was approximately symmetric and bell-shaped. Therefore, transformation of the variable was unnecessary. The Box–Cox transformation of the relative body depth with the best value of *λ* did not change the results (not shown). We calculated a neighborhood contiguity between populations (see Supplementary Figure S1) and conducted Moran’s *I* tests for residual spatial autocorrelation in linear models using R package spdep^55^ (see Supplementary Code S4). The results of non-significant Moran’s *I* tests for residuals indicate that there is little evidence of spatial autocorrelation in regression residuals. Flat semi-variograms of residuals also indicate little evidence of spatial autocorrelation in regression residuals (see Supplementary Figure S2 and Code S4). The alternative model with spatial correlation structure or sampling date as a covariate did not improve the model fitting (not shown). Therefore, we did not include the spatial correlation structure or sampling date in analyses. All datasets and R codes for the analysis were given as Supplementary Information (see Supplementary Data S1 and Code S1, S2, S3, and S4).

### Effects of gonadal condition on body depth: Pilot study 1

Sampling in different seasons (Table 1) revealed differences in the gonadal condition because the reproductive season of Japanese crucian carp is March–June in Japan. To test whether gonadal condition affects body-depth measures, or not, we measured the body depths, standard lengths, and body weights of 10 triploid mature females of Japanese crucian carp before and after natural spawning in a pilot experiment. The experimental fish were wild caught in Lake Suwa and were reared in tanks under natural light conditions for several years. The individually marked (fin-clipped) 10 females and 9 diploid males were moved to a net cage (2 × 1 × 1 m) that had been placed in a pond with spawning substrata of palm fibers on 7 May 2014. All females spawned within 3 days. The body depths of the females before (mean: 7.222 cm) and after (mean: 7.065 cm) spawning did not differ significantly (paired *t*-test, *t* = 2.066, df = 9, *P* = 0.07). In addition, the mean of their differences was small (0.157 cm). However, the body weight of the females before spawning (mean: 225.69 g) was significantly greater than that after spawning (mean: 207.63 g) (paired *t*-test, *t* = 3.99, df = 9, *P* = 0.003). Morphological observations of females before and after spawning were also performed. The females after spawning had a thin abdomen between the ventral fin and the anal fin, but showed no significant change in body shape between the pectoral fin and the ventral fin, which included the measurement site of the maximum body depth. In conclusion, results of these analyses show negligible effects of the gonadal condition on body depth.

### Gape size of predator: Pilot study 2

To determine the size at which Japanese crucian carp escape from predation by gape-limited piscivorous fish, we measured the vertical gape opening of the mouth, the mouth width, and standard length of Japanese common catfish (wild fish collected at Lake Suwa and farmed fish, 16–50 cm in standard length, *n* = 58) after anesthetization with 0.2 ml/L 2-phenoxyethanol. The vertical gape opening of the mouth was 2.2–6 cm. The mouth width was 2.6–6 cm. To predict the mouth size for a given standard length, the parameters of the allometric relation were estimated using ordinary least-squares regression (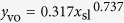, *R*^2^ = 0.86; 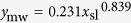, *R*^2^ = 0.94, where *x*_sl_ = standard length, *y*_vo_ = vertical gape opening, and *y*_mw_ = mouth width). Even when both variables are subject to errors, the ordinary least-squares regression is recommended for prediction (see Chapter 14.12 in an earlier report of the literature^25^). The maximum size of Japanese common catfish in Japan is about 60 cm in overall length^56^. The maximum size in standard length was estimated as about 55 cm from rough estimates of the ratio of standard to total length, 0.92 (measured from Fig. 9 in an earlier study^20^) and 0.91 (measured from Plate 60 in an earlier study^57^). Then, the vertical gape opening and the mouth width for the maximum size catfish (55 cm in standard length) were estimated respectively as 6.075 and 6.676 cm from the allometric equations. Therefore, the Japanese crucian carp larger than about 7 cm in body depth are safe from predation by the most common catfish. We did not calculate the vulnerability to predation (predation risk) using a more detailed model^58^ because it demands quantitative data that were not available (e.g., the frequency of mouth sizes in catfish population).

## Additional Information

**How to cite this article:** Kodama, S. *et al*. Allometric analysis of a morphological anti-predator trait in geographic populations of Japanese crucian carp. *Sci. Rep.* **7**, 41943; doi: 10.1038/srep41943 (2017).

**Publisher's note:** Springer Nature remains neutral with regard to jurisdictional claims in published maps and institutional affiliations.

## Supplementary Material

Supplementary Information

Supplementary Code S1

Supplementary Code S2

Supplementary Code S3

Supplementary Code S4

Supplementary Dataset

## Figures and Tables

**Table 1:** Geographic and climatic information and the sampling date of each population.

Population	Region	Latitude (°N)	Longitude (°E)	*T* (°C)	Date
A	Lake Towada	40.62	141.22	9.61	Jun 2010
B	Lake Hachirō	39.95	140.06	11.01	Apr 2010
C	Eai River	38.65	140.86	11.18	Jul 2009, Jul 2010
D	Chikuma River	36.47	138.15	11.9	Oct 2010
E	Lake Hinuma	36.29	140.53	13.76	Nov 2010
F	Lake Yanaka	36.27	139.67	14.07	Feb 2011
G	Lake Suwa	36.05	138.09	11.19	Apr–Jun 2012
H	Lake Tega	35.84	140.06	14.72	Feb 2010
I	Lake Shinji	35.45	133.06	14.96	Jan 2010
J	Hiroshiba pond	35.34	136.63	15.88	May 2010, May 2011
K	Ōtsumo River	34.84	134.60	15.32	Dec 2009, Oct 2011
L	Daibō pond	34.21	133.68	16.32	Jan 2010
M	Shimanto River	32.99	132.94	16.72	Aug 2010

Population labels follow Fig. 1. *T* = the 30-year (1984–2013) average of mean annual air temperature. Date = sampling date.

**Table 2:** Allometric slopes of body depth on standard length triploid Japanese crucian carp from 13 geographic populations.

Population	Major axis			Instrumental variable		
	Slope	95% CI	*R*^2^	Slope	95% CI	n
A	0.996	0.927–1.070	0.916	0.986	0.910–1.067	72
B	1.098	1.012–1.191	0.956	1.085	0.996–1.178	31
C	0.996	0.888–1.117	0.911	0.986	0.880–1.103	33
D	0.883	0.700–1.108	0.856	0.885	0.702–1.109	17
E	1.312	0.965–1.833	0.659	1.282	0.896–1.871	25
F	1.141	0.355–4.695	0.402	1.139	0.439–3.411	12
G	1.004	0.983–1.024	0.985	0.999	0.978–1.020	149
H	1.390	1.018–1.965	0.660	1.426	0.950–2.345	24
I	1.040	0.924–1.172	0.973	1.012	0.876–1.133	12
J	1.015	0.708–1.458	0.576	1.025	0.700–1.513	28
K	1.085	0.809–1.466	0.841	1.014	0.710–1.346	13
L	0.978	0.905–1.056	0.999	0.978	0.910–1.064	4
M	1.016	0.875–1.180	0.952	1.064	0.911–1.321	13

Population labels follow Fig. 1. Allometric slopes were obtained using the major axis and instrumental variable regression. Slope = allometric slope. 95% CI = 95% confidence intervals. *R*^2^ = coefficient of determination. *n* = number of fish sampled.
