# Erratum: Allometric analysis of a morphological anti-predator trait in geographic populations of Japanese crucian carp

**DOI:** 10.1038/srep43973

**Published:** 2017-03-14

**Authors:** Sakie Kodama, Hiroka Fujimori, Hiroshi Hakoyama

Scientific Reports 7: Article number: 4194310.1038/srep41943; published online: 02 02 2017; updated: 03 14 2017

This Article contains typographical errors in the Results section under subheading ‘Between-population and within-population variation of relative body depth’.

“The mean relative body depth of the triploid asexual Japanese crucian carp differed significantly among 13 populations (Fig. 3, Welch’s one-way ANOVA weighted for unequal variances, *F*_12,67.08_ = 26048, *P* < 2.2 × 10^−16^). The total variation in the relative body depth was 45% explained by the between-population variation and 55% by within-population variation (Kulinskaya and Staudte’s weighted coefficient of determination, *ρ*^2^ = 0.45). Within-population variation in the relative body depth was significantly different among populations (Fig. 3, Levene’s test of homogeneity of variances: *F*_12,420_ = 12.48, *P* < 2.2 × 10)”.

should read:

“The mean relative body depth of the triploid asexual Japanese crucian carp differed significantly among 13 populations (Fig. 3, Welch’s one-way ANOVA weighted for unequal variances, *F*_12,67.08_ = 26.48, *P* < 2.2 × 10^−16^). The total variation in the relative body depth was 45% explained by the between-population variation and 55% by within-population variation (Kulinskaya and Staudte’s weighted coefficient of determination, *ρ*^2^ = 0.45). Within-population variation in the relative body depth was significantly different among populations (Fig. 3, Levene’s test of homogeneity of variances: *F*_12,420_ = 12.48, *P* < 2.2 × 10^−16^)”.

