# Supplementary material for: Allometric analysis of a morphological anti-predator trait in geographic populations of Japanese crucian carp
Source: Sci Rep. 2017 Feb 2;7:41943. doi: 10.1038/srep41943 (PMC5288778; doi:10.1038/srep41943)
Supplement: Supplementary Information [file srep41943-s1.pdf]

Supplementary Information

Allometric analysis of a morphological anti-predator trait in geographic populations of Japanese crucian carp

by

Sakie Kodama, Hiroka Fujimori and Hiroshi Hakoyama

Methods

Method S1: Details of DNA ploidy measurements

The ploidy level of carp was determined by flow cytometry using a Ploidy Analyzer PA (Partec, Germany). For flow cytometry, red blood cells from living fish (ca. 0.02 ml) or fin-clips from frozen fish (ca. 5 mm^2) were sampled and immediately stored in a microfuge tube with 0.4 ml 100 % ethanol at $-30\,^{\circ}C$ until preparation. Living fish were anesthetized with 0.2 ml/L 2-phenoxyethanol. Blood samples were collected from the caudal vein using a syringe tipped with a 25G $\times$ 1" needle. The fin-clips were sampled without freeze–thaw operation to avoid cell membrane damage during thawing. The samples were prepared using a detergent solution and a staining solution. The detergent solution included 0.5 ml Tween20, 2.1 g of 0.1 M Citric acid$\cdot H_2O$ and 100 ml distilled water. The staining solution included 0.2 mg DAPI fluorochrome (4', 6-diamidino-2-phenylindole), 7.1 g $Na_2HPO_4 \cdot H_2O$ and 100 ml distilled water.[1] In cases of blood samples, a few drops of the ethanol-diluted blood (ca. 20 μl) were added to a test tube containing detergent solution 0.35 ml and incubated at room temperature ($20\,^{\circ}C$). Then, 15 min later, staining solution 1.7 ml was added to the test tube and incubated for 5 min. The DAPI fluorescence intensity of the product was measured using a flow cytometer. The intensity increases linearly with the ploidy level. In the case of fin-clips, the fin tissue was chopped with a sharp razor blade in plastic Petri dish containing detergent solution 0.35 ml and incubated for 15 min. The mixture was dropped into a test tube through a 20 μm nylon filter (CellTrics 20 μm; Partec, Germany). Staining solution 1.7 ml was added to the test tube. Then, 5 min later, the prepared sample was analyzed using flow cytometry. Flow cytometry presents the result as a peak of the DNA histogram of relative fluorescence intensity to an internal reference standard. Artificial particles (DNA control UV; Partec, Germany) and the nuclei isolated from Japanese crucian carp known ahead as diploid or triploid were used as the internal reference standard.

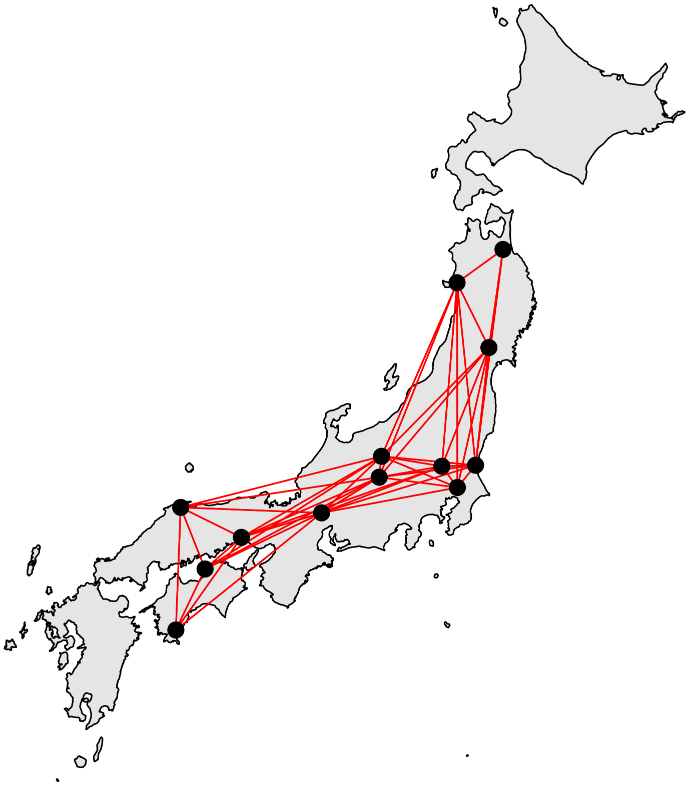

Figure S1. Graph representation of contiguity neighbors of populations. The neighborhood contiguity of populations was identified by Euclidean distance less than 500 km. Links are shown as red lines between populations (closed circles). The neighborhood contiguity was used to calculate spatial weights for Moran's *I* test for residual spatial autocorrelation (see Code S4 online). The map was constructed using R packages spdep ver. 0.6-8, maps ver. 3.1.1, and mapdata ver. 2.2-6 (URL https://cran.r-project.org/web/packages/).

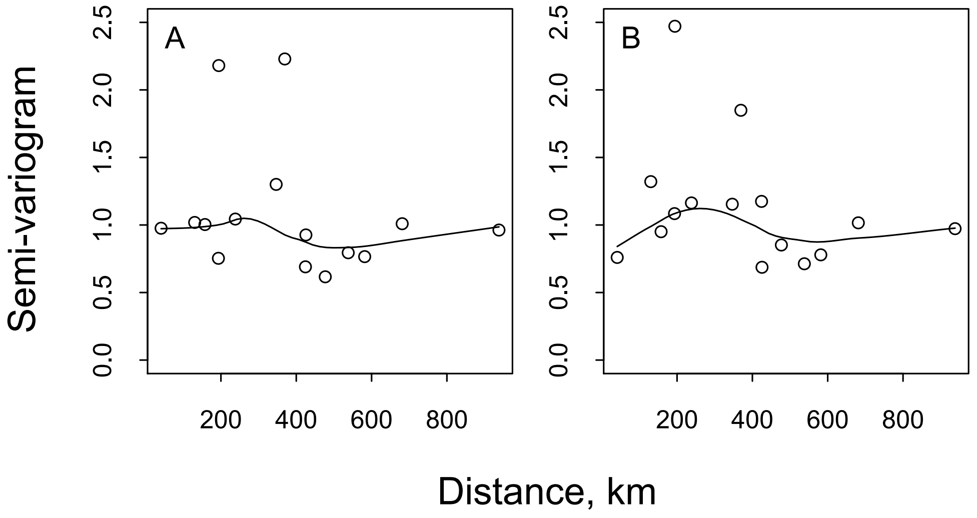

Figure S2. Experimental semi-variograms of residuals in linear models for the relative body depth of carp. Smooth curves represent the fitting results of local polynomial regression (non-parametric regression). (A) Semi-variogram of the residuals in one-way ANOVA of the relative body depth of carp among populations. (B) Semi-variogram of the residuals in a linear regression of the relative body depth on temperature. See Code S4 online.

Population	Nutrition level[2]	Area[2] (km^2)	Mean depth[2] (m)	No. of phytoplanktons[2]	No. of zooplanktons[2]	No. of fish species[2]
A	Oligotrophic	61.02	71	3	4	20
B	Eutrophic	27.73	NA	NA	NA	NA
C	NA	NA	NA	NA	NA	NA
D	NA	NA	NA	NA	NA	NA
E	Eutrophic	9.36	3	11	5	40
F	NA	4.5	NA	NA	NA	50
G	Eutrophic	12.91	4.6	9	7	16
H	Eutrophic	4.12	0.9	10	10	26
I	Eutrophic	79.08	4.5	3	8	75
J	NA	NA	NA	NA	NA	NA
K	NA	NA	NA	NA	NA	NA
L	NA	NA	NA	NA	NA	NA
M	NA	NA	NA	NA	NA	NA

Table S1. Additional information of each population. NA = Not available. An report of an earlier study[2] and unpublished information indicate that Japanese common catfish co-occur in the same habitats.

References

1. Otto, F. DAPI staining of fixed cells for high-resolution flow cytometry of nuclear DNA. *Methods Cell Biol.* **33**, 105–110 (1990).
2. Ministry of the Environment of Japan, *The 4th national survey on the natural environment.*, (1991). Available at: http://www.biodic.go.jp/reports2/4th/kosho/4_kosho_all.pdf. (Date of access: 20th December 2016).
